# Influence of Electroplated CBN Wheel Wear on Grinding Surface Morphology of Powder Metallurgy Superalloy FGH96

**DOI:** 10.3390/ma13041005

**Published:** 2020-02-23

**Authors:** Haining Wang, Xun Li, Ziming Wang, Rufeng Xu

**Affiliations:** 1 School of Mechanical Engineering, Shandong University of Technology, Zibo 255049, China; 2 School of Mechanical Engineering and Automation, Beihang University, Beijing 100191, China; wangzim@buaa.edu.cn

**Keywords:** FGH96, electroplated CBN wheel, wear, surface roughness

## Abstract

The electroplated cubic boron nitride (CBN) wheel has perfect precision retention and high-temperature grinding performance, which is widely used in the field of grinding difficult-to-cut materials, such as nickel-based superalloy. However, the research on the influence law and mechanism of grinding surface morphology affected by the wear state of small-size grinding wheel is insufficient, which limits the further promotion and application of electroplated CBN wheel in the field of precision grinding of superalloy materials. Based on the in-depth analysis of the structure of FGH96 turbine disk, and combined with the actual processing requirements, the electroplated CBN wheel was designed, and the abrasive grains size selection experiments and CBN wheel wear experiments were then carried out for the powder metallurgy superalloy FGH96 in turn. The influence law of the abrasive grain size of CBN grinding wheel and the wear state of grinding wheel on the surface morphology was investigated, respectively. The obtained results showed that under the given processing parameters, the surface roughness *R*_a_ using 400# and 600# electroplated CBN wheels are around 0.66 μm and 0.53 μm during the normal wear state of grinding wheels, respectively, which can meet the requirement of surface quality less than *R*_a_ 0.8 μm in engineering application of turbine disk, and prove the feasibility of small-size CBN wheel grinding FGH96. In addition, the failure form of electroplated CBN wheel is mainly the accumulation and adhesion of abrasive debris, and the crushing and shedding of abrasive grain are hard to appear.

## 1. Introduction

The turbine disk is the key load-bearing rotating part of aero-engine, which needs to work reliably for a long time under high temperature and pressure. Therefore, the materials of turbine disk should have excellent mechanical properties at high temperatures. At present, in order to further improve the performance of engine turbine disk, most turbine disk materials are made of nickel-based alloy. As a typical nickel-based superalloy [1], power metallurgy superalloy FGH96 has excellent high-temperature resistance and anti-fatigue performance. However, nickel-based superalloy has the characteristics of low thermal conductivity, high chemical affinity toward tool materials, and surface integrity problems [2,3,4].

Currently, in order to achieve the precision machining of difficult-to-machine materials, more and more scholars have begun to study super-hard abrasive grinding technology. Meanwhile, the electroplated cubic boron nitride (CBN) wheel has obvious advantages in grain retention and shape retention and has been widely applied [5,6]. Ding et al. [7] used brazed CBN wheel for creep feed grinding of nickel-based superalloy, the specific grinding energy of nickel-based superalloy is 200~300 J/mm^3^, and the grinding temperature is about 100 °C. Dai et al. [8] carried out experiments on grinding Inconel 718 with the vitrified CBN wheels. The results show that under the given experimental conditions, the surface roughness *R*_a_ can be less than 0.4 μm, and no distorted lattice or elongated grains are formed in the grinding surface/subsurface layer under the given experimental conditions.

However, most of these researches are limited to the traditional wheel, and in the actual machining process of turbine grooves and small holes, the traditional wheel is not easy to achieve. Recently, it has been considered feasible to use a small-diameter wheel mounted on a high-speed spindle for point grinding operation. Burrows et al. [9] carried out point grinding on Inconel 718 and Udimet 720. The experimental results show that the grinding force during finish grinding is low, the surface roughness after finish grinding is less than *R*_a_ 1.2μm, and the fatigue life of point grinding surface is longer than equivalent milled and polished specimens. As FGH96 material has not been widely used, the published research to date has not focused on point grinding of FGH96 by electroplated CBN wheel. Hence, it is of great significance to study the point grinding of CBN wheel on FGH96 for improving the surface quality of fine parts of the turbine disk and promoting the manufacture of new aero - engines.

In addition, there must be grinding wheel wear in the grinding process, which changes the grinding ability of grind grains and affects the surface quality. Therefore, it is very important to study grinding wheel wear [10]. Xu et al. [11] detected grinding wheel wear through wavelet analysis. It was found that the extracted energy ratio showed a good correlation to wheel wear state, thus achieving the purpose of monitoring grinding wheel wear. Ji et al. [12] analyzed grinding wheel wear through visual analysis. A camera was used to capture images, then image processing was executed on the images captured and, finally, the grinding layer thickness was obtained. The method could directly and quickly get the information of thickness. Benini et al. [13] specially studied the on-line detection of grinding wheel wear based on acoustic emission technology. It can be seen that using transducer to detect grinding wheel wear could effectively reduce the influence of uncertain factors caused by process parameters, workpiece, and wheel composition. Guo et al. [14] thought that power can be used as an index of grinding wheel wear. During the experiment, the power model of the grinding wheel was established by using the power data obtained with wheels worn to various stages, and the predicted results of the model were in good agreement with the measured power obtained during validation. Kaplonek et al. [15] introduced the use of focus-variation microscopy (FVM) to measure and analyze the active surfaces of a new generation of coated abrasive tools, and measured the surface of the abrasive tools through experiments, proving that FVM can be used in measurements of various abrasive tools surfaces. Most scholars regard the wear state of the grinding wheel as a goal of research, while ignoring the research on the relationship analysis between grinding wheel wear and surface quality.

In order to prove the feasibility of CBN wheel to FGH96, apply the experimental results to actual production as soon as possible, and reveal the grinding performance of small-size CBN wheels on FGH96 under different wear states, based on the actual grinding conditions, the grinding wheel was designed according to the key structure of turbine disk. The grinding performance of FGH96 was analyzed by surface roughness as the criterion of grinding wheel wear. At the same time, the wear process of electroplated CBN wheel during the grinding of FGH96 was investigated, which has great application value in the field of precision machining of FGH96 turbine disk.

## 2. Grinding Experimental Conditions

### 2.1. Electroplated CBN Wheel

**Figure 1:**
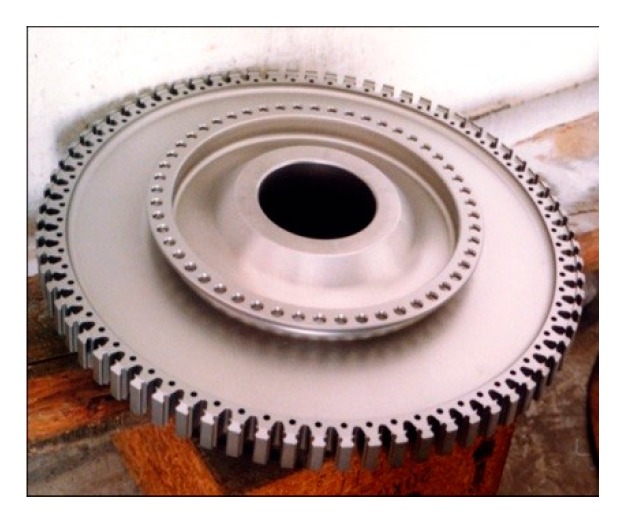
Structure of turbine disk.

The typical structural characteristics of a turbine disk are shown in Figure 1 [16,17]. Its structure is mainly a thin-walled rotating body. The disk surface has high-precision parts, such as turbine grooves, spigots, and small holes. The turbine groove at the outer edge is composed of a linear segment, concave arc, and convex arc. The radius of the arc is generally R0.4 mm ~ R2 mm, the thickness of the spigots is generally 4.5mm, and the radius of the hole is less than R5mm.

**Figure 2:**
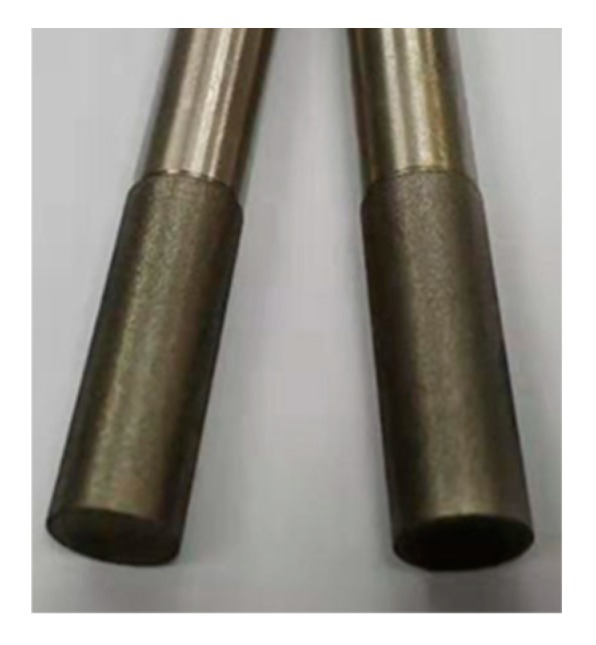
Electroplated cubic boron nitride (CBN) wheel.

The contour accuracy and surface quality of each precise part on the turbine disk need to be guaranteed. In addition, the surface roughness of precise parts should be lower than *R*_a_ 0.8 μm [18]. In order to explore the influence of grinding technology on the surface quality and provide experimental reference for the grinding of each fine part, a corresponding grinding wheel was designed for grinding test study of FGH96 according to the structure characteristics of turbine grooves, spigots, and small holes on the turbine disk, as shown in Figure 2.

The diameter of the grinding wheel is Φ6 mm, and the length of grinding part is 18 mm. A small groove is set at the connection between the working part of the grinding wheel and the clamping part to prevent the accuracy of the clamping part from being affected during electroplating.

FGH96 is a kind of difficult-to-cut material. The grinding grains of wheel should have high strength, hardness, and high temperature resistance. Studies have shown that CBN electroplated wheel has good profile retention and particle retention, which is widely used in machining super difficult-to-cut materials [18,19].

### 2.2. Experimental Equipment

**Figure 3:**
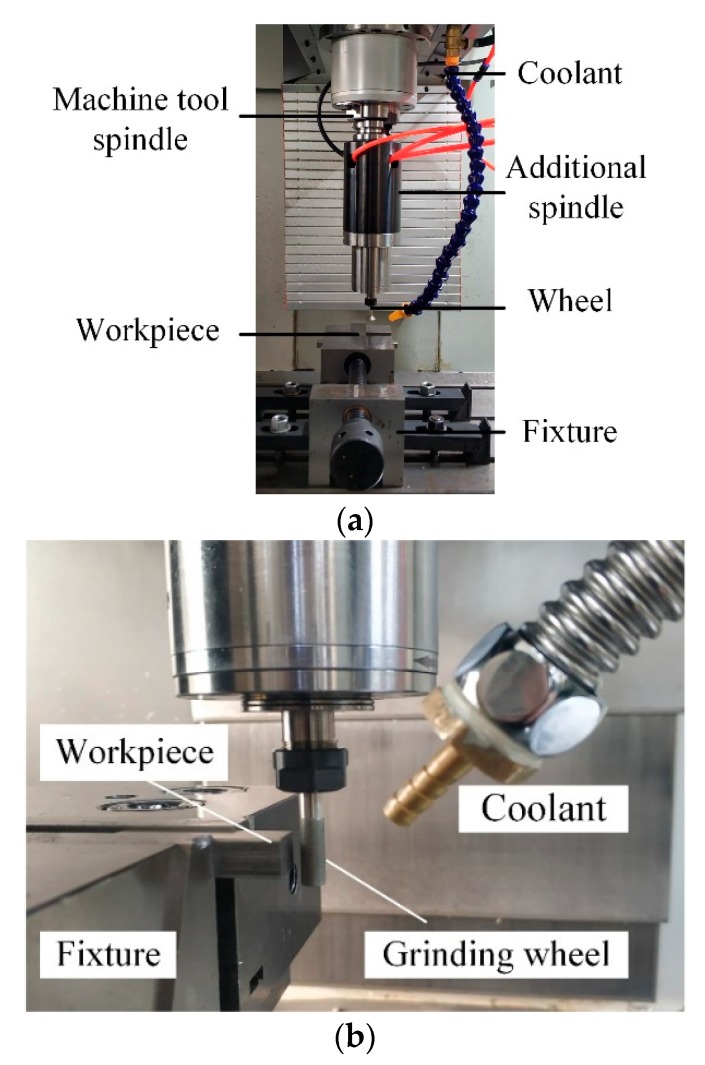
Additional high-speed spindle. (**a**) High-speed spindle, (**b**) Machining setup.

Due to the small diameter of the wheel, the high-speed spindle is needed to meet the requirement of grinding linear speed, which is a very important grinding condition. In order to achieve the required linear speed, an additional high-speed spindle is utilized, as shown in Figure 3.

The maximum speed of the additional high-speed spindle is 90,000 rpm, and the speed is adjusted by a converter. The water cooler is used to keep the temperature of the high-speed spindle within the normal range.

The experiment mainly studied the surface roughness of workpiece affected by the wear and the morphology of the wheel surface. The surface roughness of the workpiece perpendicular to the feed speed direction was measured by a TIME3220 roughness tester (Beijing Times High Technology Co., Ltd., Beijing, China). The surface morphology of wheel was examined by a scanning electron microscope (SEM) (FEI, Hillsboro, OR, USA).

### 2.3. Experimental Materials of Workpiece

**Figure 4:**
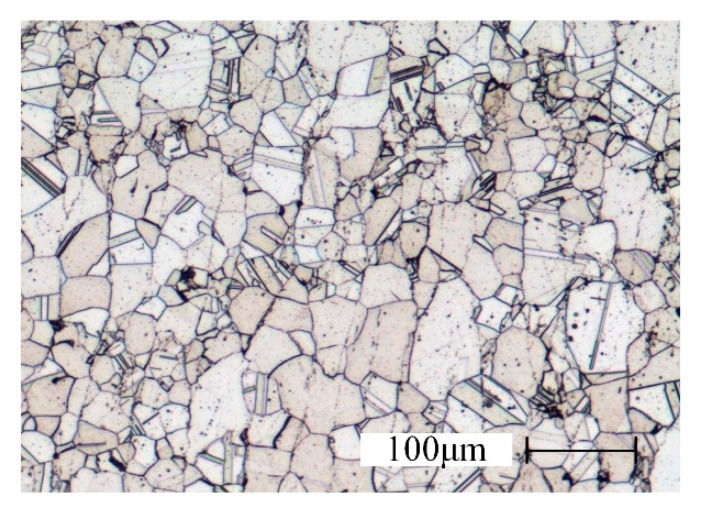
Microstructure of FGH96.

The test material is powder metallurgy superalloy FGH96. Its working temperature can reach 765 °C, and it has good crack growth resistance. It can completely meet the requirements of high temperature and high thrust-to-weight ratio for high-performance aero-engines [20]. The main components are shown in Table 1 [21,22], and the internal organization of FGH96 is shown in Figure 4.

The grain size of FGH96 is up to 150 μm, and the hardness is about 419 HV. The dimensions of the test workpiece are 30 × 15 × 8 mm. The compressing grinding mode is utilized in all experiments. Cimcool Cimtech 310 cooling fluid (Cimcool Industrial Products (Shanghai) Co., Ltd., Shanghai, China) is used for cooling.

**Figure 5:**
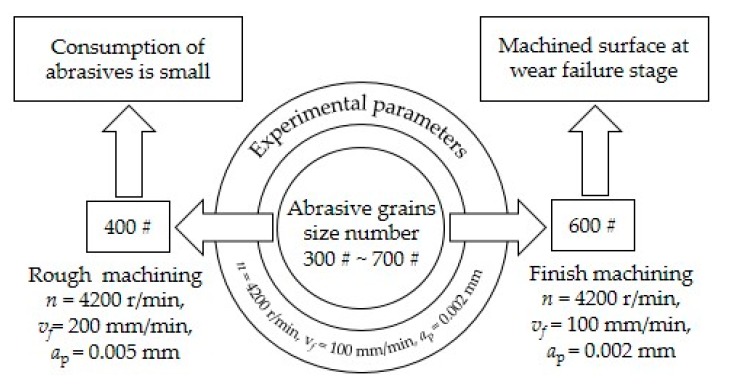
Graphic abstract of grinding experiment.

In this study, firstly, surface roughness of FGH96 with different grains size number wheels was analyzed. According to the actual production requirements, it is found that 400 # grinding wheel is suitable for rough machining and 600 # grinding wheel is suited to finish machining. Then, the surface morphology of the two wheels and workpiece after grinding is analyzed in detail, and the experimental process is shown in Figure 5.

## 3. Effect of Grain Size of Electroplated CBN Wheel on Surface Roughness

**Figure 6:**
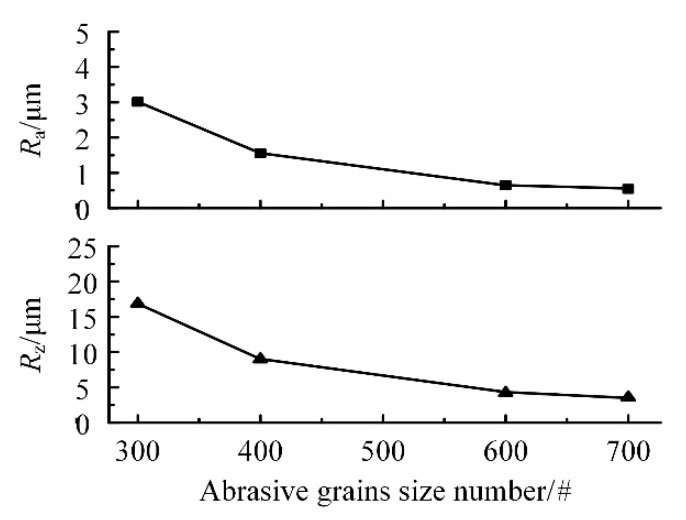
Effect of abrasive grains size on surface roughness.

During the grinding process, abrasive grain size has a great influence on the ground surface roughness. Therefore, it is necessary to obtain the influence law of the abrasive grain size on the surface roughness of FGH96. The unworn grinding wheel with different abrasive grain sizes was used for FGH96 grinding experiment. The experimental parameters were *n* = 42,000 r/min, *v_f_* = 100 mm/min, and *a*_p_ = 0.002 mm. Other conditions were guaranteed to remain unchanged during the grinding process. The measurement results of the grinding surface roughness are shown in Figure 6.

According to the experimental results, the surface roughness machined by 300# CBN wheel is about *R*_a_ 3.01 μm and *R*_z_ 16.87 μm. The surface roughness machined by 700# CBN wheel is less than *R*_a_ 0.54 μm and *R*_z_ 4.03 μm. Meanwhile, both *R*_a_ and *R*_z_ decrease with the increase of abrasive grain size number. Therefore, the change of abrasive grain size has a significant impact on the surface roughness, which is dependent on the manufacturing process of the grinding wheel. The arrangement of the abrasive grains on the surface of wheel is random. Different grinding wheels have different abrasive grain sizes. When the abrasive grain size number is large, the size of abrasive grains is small and the number of abrasive grains involved in the grinding is large, which can make the machined surface smooth. Therefore, under the same processing parameters, the grinding process utilizing a large grain size number has less material removal and shallower scratches, which will result in a smaller surface roughness value.

## 4. Effect of Electroplated CBN Wheel Wear on Grinding Surface

### 4.1. Effect of Grinding Wheel Wear State on Surface Roughness

Grinding wheel wear generally goes through three stages, including initial wear, normal wear, and wear failure [23]. In the experiment, different variation trends of surface roughness are used to distinguish the wear stages of the wheel. In addition, an important purpose of machining is to obtain the required surface topography, and the most direct evaluation criterion is surface roughness. Therefore, it has great significance to take the grinding surface roughness as the evaluation standard of wheel wear.

**Figure 7:**
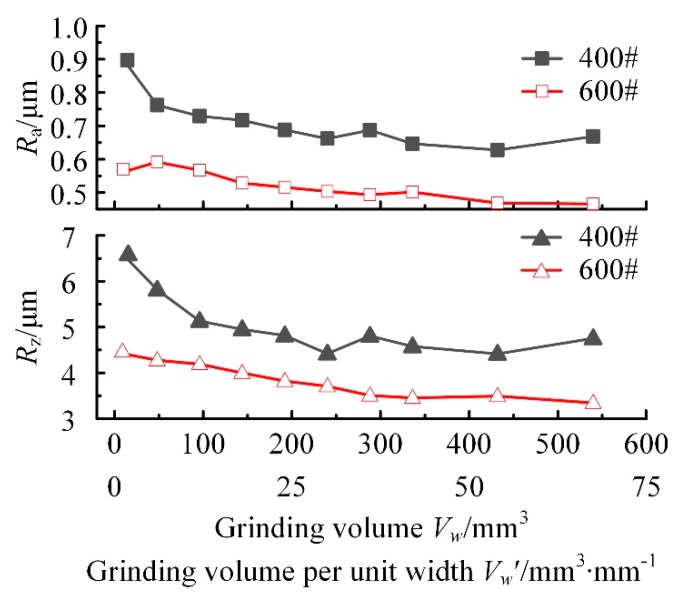
Effect of grinding wheel wear on surface roughness.

Generally, the surface roughness of the initial wear stage decreases rapidly, which is mainly formed by the crushing and abrasion of the abrasive grains. The surface roughness of the normal wear stage can remain stable, but the abrasive grains become less sharp. The value of surface roughness in the wear failure stage is relatively small, and the grinding grains will fall off after grinding, accompanied by the occurrence of chatter trace and other defects [16]. According to the surface quality requirements of precision parts and the effect of abrasive grain size number on the surface roughness, wear experiments of 400# and 600# grinding wheels were carried out, respectively. Considering that the size of the abrasive grain is different, the abrasion resistance will also be different. Therefore, in order to improve the experimental efficiency, the grinding depth, feed speed, and other grinding parameters were selected. The processing parameters of 400# CBN wheel were *n* = 42,000 r/min, *v_f_* = 200 mm/min, and *a*_p_ = 0.005 mm and 600# CBN wheel were *n* = 42000 r/min, *v_f_* = 100 mm/min, and *a*_p_ = 0.002 mm. The experimental results are shown in Figure 7.

The overall tendency of surface roughness *R*_a_ machined by CBN wheel is reduced with the increase of the grinding amount. When the grinding amount *V*_w_ accumulates from 0 to 450 mm^3^ (the grinding volume per unit width reaches 56.25 mm^3^∙mm^−1^), the surface roughness of 400# CBN wheel decreases from *R*_a_ 0.90 μm to *R*_a_ 0.65 μm. During the grinding process, when the grinding volume *V*_w_ reaches 100mm^3^ (*V*_w_′ = 12.5 mm^3^∙mm^−1^), the surface roughness decreased to *R*_a_ 0.73 μm. Moreover, from this point, the surface roughness began to stabilize. The slope of roughness value *R*_a_ machined by 600# CBN wheel changes little with the increasing of grinding amount, and the change of *R*_a_ value is also small. When the grinding amount *V*_w_ accumulates from 0 to 50 mm^3^ (*V*_w_′ = 6.25 mm^3^∙mm^−1^), the surface roughness is about *R*_a_ 0.56 μm stably.

During the grinding process, surface roughness *R*_z_ and *R*_a_ have the same change trends. When grinding volume accumulates to *V*_w_ = 100 mm^3^ (grinding volume per unit width *V*_w_′ = 12.5 mm^3^∙ mm^−1^), the surface roughness *R*_z_ of 400# CBN wheel decreases from 6.54 μm to 5.13 μm, which indicates that the height of abrasive grains changes greatly at the initial grinding stage, meanwhile, the wear resistance of abrasive grains gradually appears. The slope of *R*_z_ machined by 600# CBN wheel remains almost unchanged, and the change of *R*_z_ value is also very small. When *V*_w_′ cumulative exceeds 50 mm^3^ (*V*_w_′ = 6.25 mm^3^ mm^−1^), the surface roughness is about *R*_z_4.27 μm from *R*_z_4.43 μm by the wheel with initial grinding.

A comprehensive analysis was performed on the change trends of *R*_a_ and *R*_z_ and take *V*_w_ = 100 mm^3^ (*V*_w_′ = 12.5 mm^3^∙mm^−1^) as the boundary between the initial wear stage and the normal wear stage of 400# CBN wheel. During the normal wear stage of 400# CBN wheel, the roughness *R*_a_ is about 0.66 μm and *R*_z_ is about 4.75 μm stably. The 400# CBN wheel requires a large grinding amount to achieve the normal wear state, and the value of surface roughness changes significantly in the initial wear stage compared to the normal wear stage. It is shown that the size of abrasive grains is larger and sharper in the initial wear stage of wheel, and the ability to scratch the surface is stronger. In the later normal wear stage of wheel, the sharpness of abrasive grains is reduced, which results in the decrease of surface roughness. *V*_w_ = 50 mm^3^ (*V*_w_′ = 6.25 mm^3^∙mm^−1^) is taken as the boundary between the initial wear stage and the normal wear stage of 600# CBN wheel. During the normal wear stage of 600# CBN wheel, the roughness *R*_a_ is about 0.53 μm and *R*_z_ is about 3.76 μm stably. With the increase of grinding amount, the changes of *R*_a_ and *R*_z_ are not obvious, which indicates that the grinding amount of 600# CBN wheel with the initial wear is small, and the boundary between the initial wear stage and the normal wear stage is not obvious under the given grinding parameters.

### 4.2. Surface of Electroplated CBN Wheel

#### 4.2.1. Inspection of the Wheel Surface with Normal Wear

**Figure 8:**
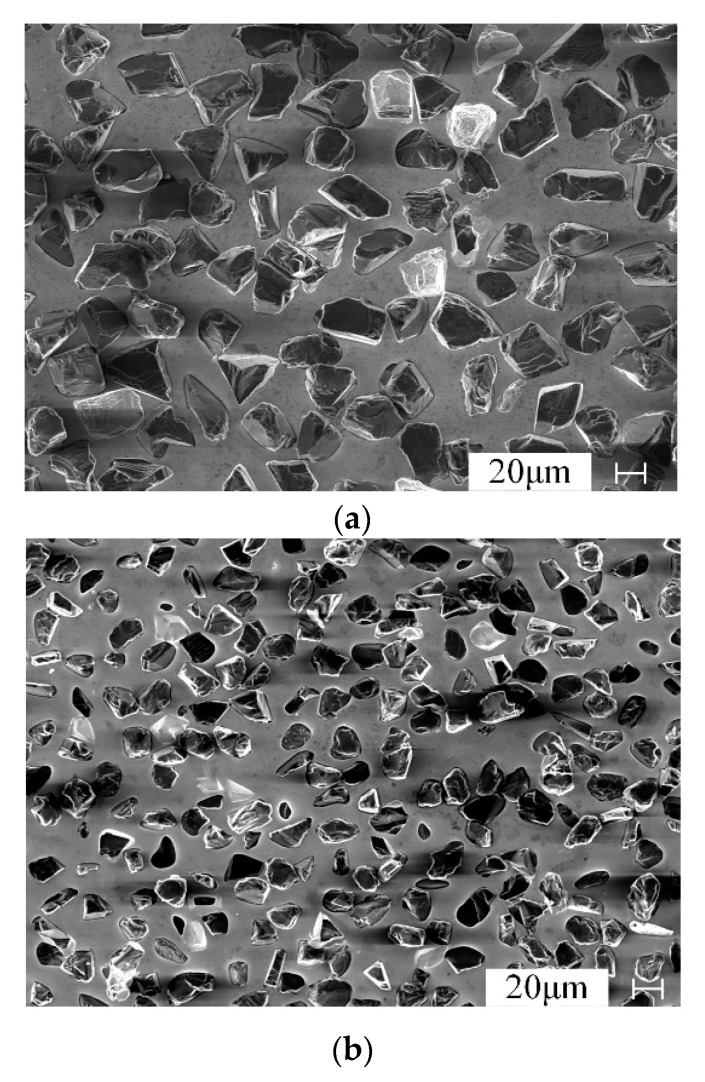
Surface morphology of the CBN wheel with normal wear. (**a**) Surface morphology of 400# CBN wheel, (**b**) Surface morphology of 600# CBN wheel.

The grinding wheel with normal wear is observed by SEM, and the obtained results are shown in Figure 8.

In Figure 8, it can be seen that the grinding grains on the wheel surface are randomly distributed. During the normal wear stage, no obvious abrasive grains fall off from the wheel surface.

The abrasive grains are constantly worn out, and the new abrasive grains are constantly protruding, which belongs to the self-sharpening stage of CBN wheel. It can be indicated that both types of CBN wheels have perfect abrasive grains retention under the given grinding parameters. From the above results, it can be concluded that the electroplated CBN wheel is completely suitable for grinding FGH96.

#### 4.2.2. Inspection of the Wheel Surface with Wear Failure

**Figure 9:**
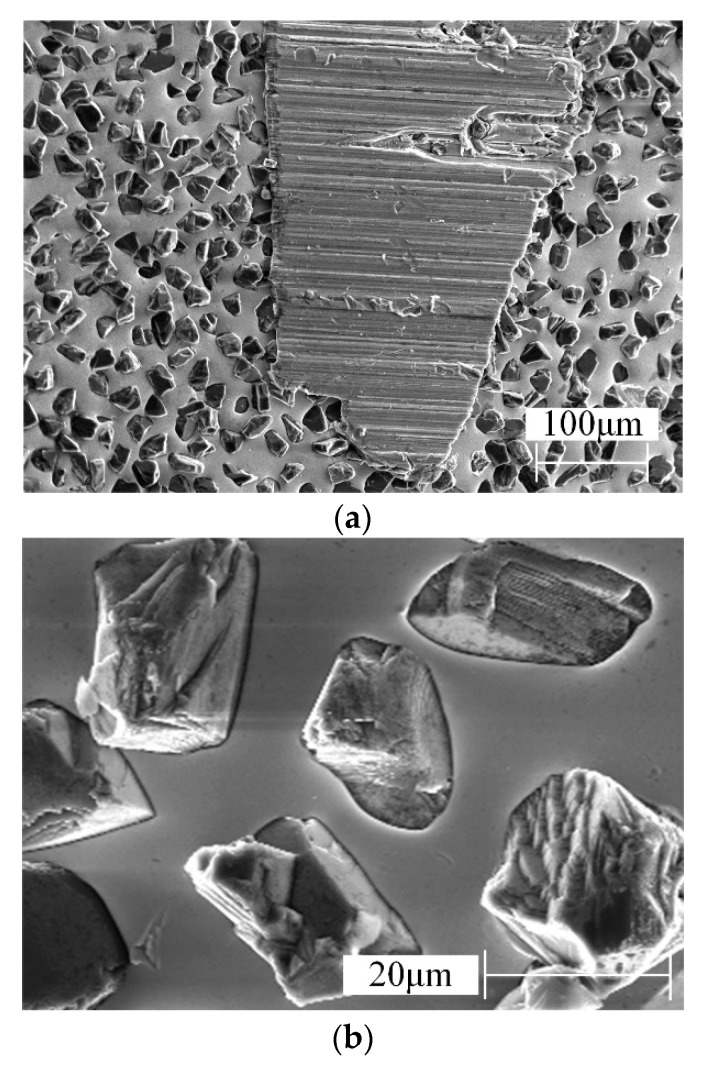
Surface of 600# CBN wheel with wear failure by SEM. (**a**) Morphology of the wheel, (**b**) Abrasive grains on the wheel surface.

Due to the large abrasive grains of 400# CBN wheel, the consumption of abrasive grains is small during the normal wear stage. When the total grinding volume *V*_w_ exceeds 550 mm^3^ (*V_w_*′ = 68.75 mm^3^∙mm^−1^), the wheel has an excellent grinding performance stably and normally, which can fully meet the engineering application of turbine groove grinding. However, when *V*_w_ of 600# CBN wheel exceeds 426 mm^3^ (*V_w_*′ = 53.25 mm^3^∙mm^−1^), chatter trace is generated on the machined surface and the roughness decreases to *R*_a_ 0.46 μm and *R*_z_ 3.49 μm, which can be regarded as the beginning of wear failure stage. At the same time, the surface and abrasive grains of 600# CBN wheel with wear failure were observed by SEM, as shown in Figure 9.

In the wear failure stage, there are not many abrasive grains falling off from the wheel surface and the consumption of grains is small, while the abrasive debris accumulation phenomenon occurs on the wheel surface. The accumulation layer with the width of about 270 μm completely covers the abrasive grains, and scratches appear on the surface, indicating that there is an obvious extrusion process, which is a blockage phenomenon of CBN wheel. Comprehensive analysis from Figure 9 indicates that the poor surface quality machined by the wheel with wear failure is not caused by the shedding or breaking of abrasive grains but by the accumulation of abrasive debris in the gaps among the abrasive grains.

### 4.3. Effect of the Wheel Wear on the Machined Surface

**Figure 10:**
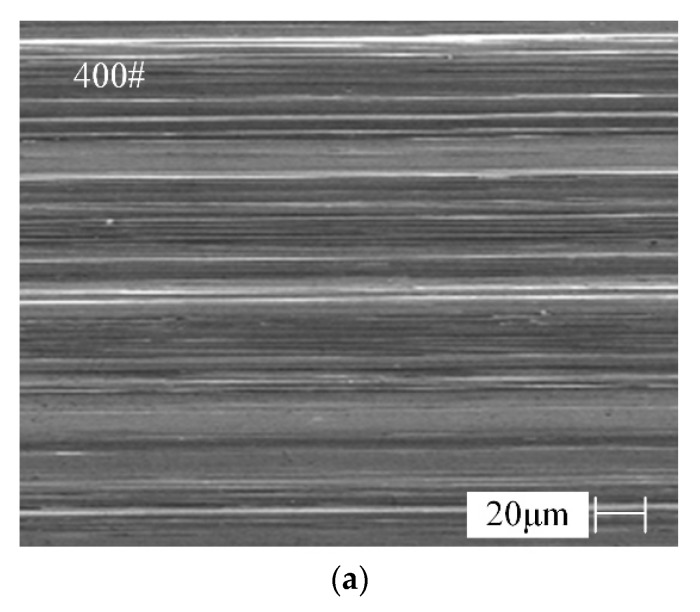

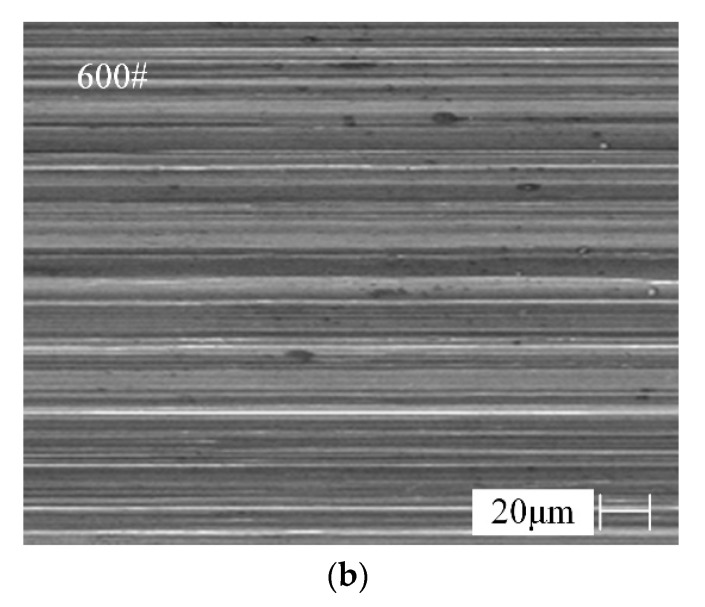
Surface topography by the different types of CBN wheel with normal wear. (**a**) Surface morphology by 400# CBN wheel with normal wear, (**b**) Surface morphology by 600# CBN wheel with normal wear.

The machined surface topographies by 400# and 600# CBN wheel with normal wear are shown in Figure 10.

As can be seen from Figure 10, the scratches generated are different in depth and uneven in distribution. There are more deep scratches on the machined surface by 400# CBN wheel and distribution interval of scratches is large, resulting in the larger value of surface roughness. The distribution of deep and shallow scratches on the machined surface by 600# CBN wheel is relatively uniform and smooth, which can indicate that the height difference of 600# abrasive grain is small. There is no obvious defect on the grinding surface, which indicates that the grinding process is stable.

**Figure 11:**
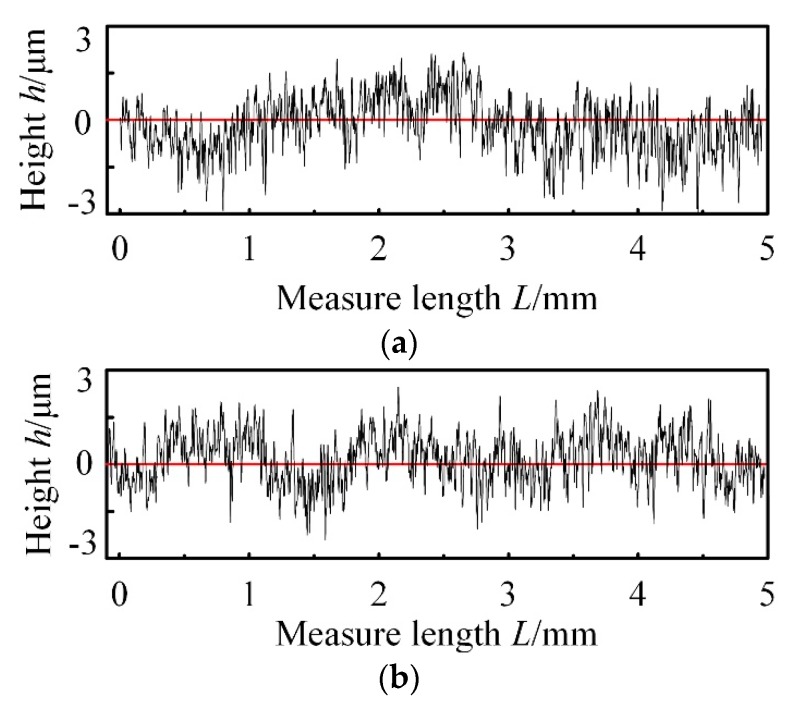
Surface profiles by different types of CBN wheels with normal wear. (**a**) Grinding surface profile by 400# CBN wheel, (**b**) Grinding surface profile by 600# CBN wheel.

The roughness tester was used to measure the surface profile perpendicular to the feed direction, and the original data are shown in Figure 11.

As can be seen from Figure 11, the height difference of 400# CBN wheel and 600# CBN wheel is less than 6 μm, while surface roughness value by 600# wheel is smaller than 400# wheel. This is because of different arrangement and size of abrasive grains, which makes height - point intervals by 600# CBN wheel is regular than that by 400# CBN wheel.

**Figure 12:**
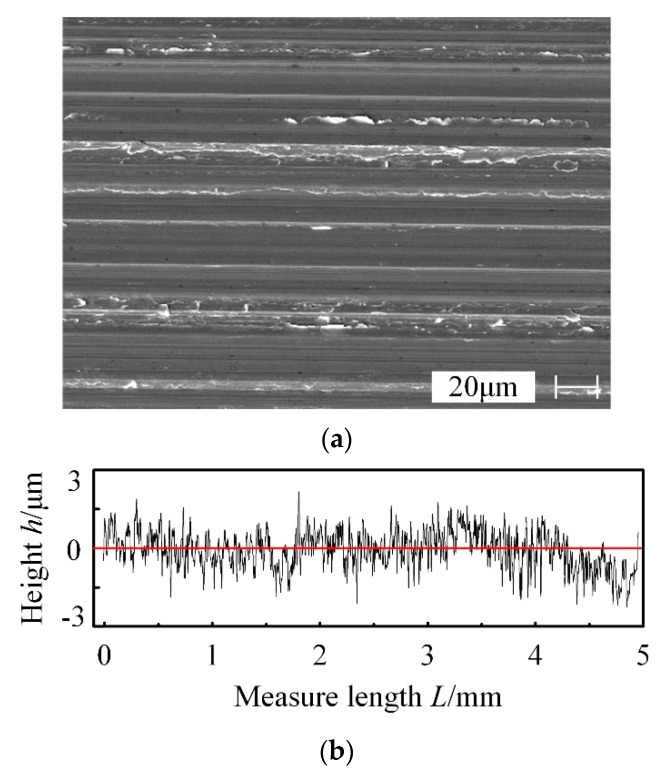
Machined surface by the 600# CBN wheel with wear failure. (**a**) Surface morphology machined by CBN wheel with failure wear, (**b**) Profile at the wear failure stage.

The machined surface morphology and profile by 600# CBN wheel with wear failure are shown in Figure 12.

From Figure 12a, a lot of burrs and scales can be seen on the grinding surface due to extrusion, but the surface scratches are not clear, which reduce the finished surface quality seriously. From Figure 12b, the curve of surface profile varies within a smaller range compared with that machined by the CBN wheel with normal wear, and there are no large valley peaks and valleys, indicating that abrasive grinding process is changing to wheel extrusion process, which reduce the roughness value. Meanwhile, the significant defects will have a negative effect on the fatigue performance of FGH96 part. Hence, the grinding volume *V*_w_′ of CBN wheel should be controlled strictly and cannot exceed 80 mm^3^∙mm^−1^. The grinding volumes are completely sufficient for the finishing process, which indicates that 600# CBN wheel is suitable for the finishing process of FGH96.

## 5. Conclusions and Future Development

Based on the actual industrial case of aero-engine parts manufacturing, we have studied the small-size CBN wheel grinding effect on powder metallurgy superalloy FGH96. The feasibility of grinding FGH96 by small-size CBN wheel tools in different wear states has been proved, which can, indeed, provide the reference for machining of turbine discs made of FGH96 material. Furthermore, the following experimental results have been obtained:

1. Precision grinding of powder metallurgy superalloy FGH96 can be realized by small-size electroplated CBN wheel. According to the variation trend of roughness, grinding wheel wear is divided into three stages: (1) initial wear, (2) normal wear, and (3) wear failure, which can provide a reference for studying the wheel wear.
2. In the initial wear stage of CBN wheel, with the grain size number of the grinding wheel increasing from 300# to 700#, the grain size decreases, the surface scratches become shallower, and the surface becomes smoother, which make the grinding surface roughness of FGH96 decrease from *R*_a_ 3.01 μm to *R*_a_ 0.54 μm.
3. In the normal wear stage of CBN wheel, there are no defects on the surface after machining, but uneven deep scratches will occur, and with the increase of abrasive grain size number, the wear resistance of electroplated CBN wheel gradually decreases. Within the range of experimental parameters, 400# electroplated CBN wheel enters the normal wear stage from the grinding volume per unit width *V*_w_′ = 12.5 mm^3^∙mm^−1^, and the deep scratching phenomenon is obvious, while 600# electroplated CBN wheel enters the normal wear state from the grinding volume per unit width *V*_w_′ = 6.25 mm^3^∙mm^−1^, and the surface is more uniform. At the same time, both of the grinding surface roughness is *R*_a_ 0.73 μm and *R*_a_ 0.56 μm, respectively. This shows that 400# CBN wheel has good wear resistance, while 600# CBN wheel is slightly weaker.
4. During the process of grinding FGH96 with electroplated CBN wheel, CBN wheels have perfect abrasive grain retention, meanwhile, the profile shape and grinding performance of CBN abrasive grains are well maintained. In the failure wear stage, the failure form of CBN wheel is mainly debris accumulation and adhesion on the wheel surface, which makes the wheel blunt. Under the given experimental conditions, when the total grinding quantity *V*_w_ of 600# grinding wheel exceeds 426 mm^3^ (grinding volume per unit width *V*_w_′ = 53.25 mm^3^∙mm^−1^), the grinding performance of the grinding wheel decreases obviously and scales appear on the surface of grinding workpiece. This indicates that 600# CBN wheel can only be used for finishing process.

This work can be directly applied to machining of small holes and structures in turbine disks. In the future, the aviation industry will be further developed, the precision machining of turbine disks will face challenges, and small profiling grinding wheels will play an important role. Next, we will further investigate the surface quality and service life of FGH96 after machining and, based on the current research and according to the shape of turbine groove, design a small-size profile CBN wheel. 

## Figures and Tables

**Table 1:** Components of FGH96.

C	Cr	Co	W	Mo	Al	Ti	Nb	Zr	Ni
0.02~0.05	15.5~16.5	12.5~13.5	3.8~4.2	3.8~4.2	2.0~2.4	3.5~3.9	0.6~1.0	0.025~0.05	Bal.

## References

[1] Xu J.H., Zhang Z.W., Fu Y.C. (2014). Review and Prospect on High Efficiency Profile Grinding of Nickel-based Superalloys. Acta. Aeronaut Astronaut. Sin..

[2] Thakur A., Gangopadhyay S. (2016). State-of-the-art in surface integrity in machining of nickel-based super alloys. Int. J. Mach. Tools Manuf..

[3] Zhu D.H., Zhang X.M., Ding H. (2013). Tool wear characteristics in machining of nickel-based superalloys. Int. J. Mach. Tools Manuf..

[4] Miao Q., Ding W.F., Kuang W.J., Yang C.Y. (2020). Comparison on grindability and surface integrity in creep feed grinding of GH4169, K403, DZ408 and DD6 nickel-based superalloys. J. Manuf. Process.

[5] Hitchiner M.P., McSpadden S.B., Webster J.A. (2005). Evaluation of factors controlling CBN abrasive selection for vitrified bonded wheels. CIRP Ann..

[6] Ding W.F., Linke B., Zhu Y.J., Li Z., Fu Y.C., Su H.H. (2016). Review on monolayer CBN superabrasive wheels for grinding metallic materials. Chin. J. Aeronaut..

[7] Ding W.F., Xu J.H., Chen Z.Z., Su H.H., Fu Y. (2010). Grindability and Surface Integrity of Cast Nickel-based Superalloy in Creep Feed Grinding with Brazed CBN Abrasive Wheels. Chin. J. Aeronaut..

[8] Dai C.W., Ding W.F., Zhu J.Y., Xu J.H., Yu H.W. (2018). Grinding temperature and power consumption in high speed grinding of Inconel 718 nickel-based superalloy with a vitrified CBN wheel. Precis. Eng..

[9] Burrows J.M., Dewes R.C., Aspinwall D.K. Grinding of Inconel 718 and Udimet 720 using superabrasive grinding points mounted on a high speed machining centre. Proceedings of the 33rd International MATADOR Conference.

[10] Xiang D.H., Zhou Z.K., Liu Z.Y., Yao Y.L., Guo Z.H. (2018). Abrasive wear of a single CBN grain in ultrasonic-assisted high-speed grinding. Int. J. Adv. Manuf. Tech..

[11] Xu L.M., Xu K.Z., Chai Y.D. (2010). Identification of grinding wheel wear signature by a wavelet packet decomposition method. J. Shanghai Jiaotong Univ. (Sci.).

[12] Ji Y.C., Fu L.H., Yang D.J., Wang L. A method of detection to the grinding wheel layer thickness based on computer vision. Proceedings of the 2017 International Conference on Optical Instruments and Technology: Optoelectronic Measurement Technology and Systems.

[13] Benini L., Weingaertner W.L., Lucas D.S.M. (2014). Wear Monitoring on Microcrystalline Aluminum Oxide Grinding Wheels on Profile Grinding with the Aid of Acoustic Emission. Adv. Mater. Res..

[14] Guo C., Shi Z., Attia H., Mclntosh D. (2007). Power and Wheel Wear for Grinding Nickel Alloy with Plated CBN Wheels. CIRP Ann.—Manuf. Techn..

[15] Kaplonek W., Nadolny K., Królczyk G.M. (2016). The Use of Focus-Variation Microscopy for the Assessment of Active Surfaces of a New Generation of Coated Abrasive Tools. Meas. Sci. Rev..

[16] Zhou W.L., Chen W.H., Zhang F.J. (2014). Forming Process Simulation and Optimization of Nickel-Base Superalloy Turbine Disk. Adv. Mater. Res..

[17] Chen Q., Guo H., Zhang C., Liu X. (2014). Structural optimization of uniaxial symmetry non-circular bolt clearance hole on turbine disk. Chin. J. Aeronaut..

[18] Shi Z.D., Elfizy A., Pierre B.S., Attia H. (2012). Grinding characteristics of a nickel-based alloy using vitrified CBN wheels. Int. J. Abras. Technol..

[19] Chen Z.Z., Xu J.H., Ding W.F., Ma C.Y. (2014). Grinding performance evaluation of porous composite-bonded CBN wheels for Inconel 718. Chin. J. Aeronaut..

[20] Handbook of Chinese Aerial Materials Editorial Committee (2002). The Handbook of Chinese Aerial Materials.

[21] Ma T.J., Wang W., Li W.Y., Zhang Y. (2014). Microstructure and mechanical properties of linear friction welded P/M superalloy FGH96. J. Mater. Eng..

[22] Liu J., Liu G.Q., Hu B.F., Song Y.P., Qin Z., Zhang Y.W. (2006). Hot deformation behavior of FGH96 superalloys. J. Univ. Sci. Technol. Beijing Miner. Metall. Mater..

[23] Li B.M., Zhao B. (2003). Modern Grinding Technology.

